# Implementation and evaluation of a Tele-OSCE in oral and maxillofacial surgery – a pilot report

**DOI:** 10.3205/zma001571

**Published:** 2022-11-15

**Authors:** Lukas Benedikt Seifert, Alawia Coppola, Julian Wilhelm Amadeus Diers, Christoph Kohl, Vanessa Britz, Jasmina Sterz, Miriam Rüsseler, Robert Sader

**Affiliations:** 1 University Hospital Frankfurt, Department of Oral, Cranio-Maxillofacial and Facial Plastic Surgery, Frankfurt, Germany; 2 Goethe University, Medical Faculty, Frankfurt Interdisciplinary Simulation Center (FIneST), Frankfurt, Germany; 3 University Hospital Frankfurt, Department for Trauma, Hand and Reconstructive Surgery, Frankfurt, Germany

**Keywords:** oral and maxillofacial Surgery, dentoalveolar surgery, telemedicine, teledentistry, OSCE

## Abstract

**Background:**

The ongoing changes in learning and education towards digitalisation have been rapidly accelerated by the COVID-19 pandemic. Especially in dental education where contact to the oral cavity is an integral part of training the chosen digital examination methods and training formats must undergo high requirements to full fill the goal of a real alternative to face-to-face exams. Therefore, this study compared student performance in a newly developed Tele-OSCE with a prior OSCE examinations in presence within an oral- and maxillofacial surgery curriculum.

**Methods:**

Study participants were fourth-year (in a five year curriculum) dental students and board certified maxillofacial surgeons (examiners) that took part in a newly developed Tele-OSCE that comprised three five-minute stations (structured facial examination, management mandibular fracture and squamous cell carcinoma) using the zoom® software. Student performance was measured using validated OSCE-Checklists and compared to a previous OSCE examination from the winter term 2019 with the same OSCE stations that was conducted in presence. Significant differences were tested using the Mann-Whitney U test. Furthermore, the new Tele-OSCE was evaluated by students and examiners using previously developed questionnaires.

**Results:**

Sixty-six dental students (study group: n=34, summer term 2021, control group: n=32 winter term 2019) and nine examiners participated in the study. Compared to previous non-pandemic OSCEs, there were no significant (p=0.53) differences in overall student performance. Evaluation of the Tele-OSCE showed that the demonstration and rating of practical skills was limited due to missing standard patients or phantoms, however, students did not fear to be misjudged. The demonstration and rating of anamnestic and consultation competencies was seen as unproblematic by students and examiners.

**Discussion:**

This pilot-study showed the feasibility of a Tele-OSCE as a formative examination in dental education. However, both students and examiners felt that the demonstration and assessment of practical skills was limited due the new examination format. Nevertheless, Tele-OSCEs might offer an alternative to enable students to complete their dental training.

## Zusammenfassung

**Hintergrund:**

Der stetige Wandel hin zur Digitalisierung im Bildungswesen wurde durch die Covid-19 Pandemie rasant beschleunigt. In der zahnmedizinischen Ausbildung, wo der Kontakt zur Mundhöhle integraler Bestandteil der klinischen Lehre ist, müssen die gewählten digitalen Lehrformate jedoch hohen Anforderungen unterliegen, um eine sinnvolle Alternative zur Präsenzlehre darzustellen. Ziel dieser Pilotstudie war deshalb einen neu entwickelten Tele-OSCE in der Mund-, Kiefer- und Gesichtschirurgie mit vorherigen Präsenz-OSCE Prüfungen hinsichtlich Performanz und Zufriedenheit bei Studierenden und Prüfern zu evaluieren.

**Methoden:**

Die Studienteilnehmer waren Zahnmedizinstudierende im vierten Jahr und MKG-ChirurgInnen (Prüfer), die an einer neu entwickelten Tele-OSCE teilnahmen, welche drei fünfminütige Stationen umfasste (1. strukturierte Gesichtsuntersuchung, 2. Management von Unterkieferfrakturen, 3. Management des Plattenepithelkarzinoms). Der Kontakt zwischen Studierendem und Prüfer wurde mit der Zoom®-Software hergestellt. Die studentischen Leistungen wurden anhand validierter OSCE-Checklisten gemessen und mit einer vorangegangenen Präsenz-Kohorte aus dem Wintersemester 2019 verglichen. Die Leistung der Studierenden wurde mittels Mann-Whitney-U-Test auf signifikante Unterschiede getestet. Darüber hinaus wurde das neue Prüfungsformates durch Studierende und Prüfer anhand zuvor entwickelter Fragebögen evaluiert.

**Ergebnisse:**

An der Studie nahmen 66 Studierende der Zahnmedizin (Studiengruppe: n=34, Sommersemester 2021, Kontrollgruppe: n=32 Wintersemester 2019) und neun PrüferInnen teil. Im Vergleich zur Präsenz-Kohorte von 2019 gab es keine signifikanten (p=0,53) Unterschiede in der Gesamtleistung der Studierenden. Die Evaluation zeigte jedoch, dass die Demonstration und Bewertung praktischer Fertigkeiten im Tele-Setting nur eingeschränkt möglich waren. Eine Angst schlechter oder falsch bewertet zu werden bestand auf Studierendenseite nicht. Der Demonstration und Bewertung von anamnestischen und beratenden Kompetenzen wurden von Studierenden und Prüfern als unproblematisch eingeschätzt.

**Diskussion:**

Diese Pilotstudie konnte zeigen, dass eine Tele-OSCE in der zahnmedizinischen Ausbildung als Alternative zu Präsenzprüfungen problemlos implementierbar ist und zu keinen Leistungsunterschieden in Vergleich zu Präsenzprüfungen führt. Allerdings empfanden sowohl Studierende als auch PrüferInnen die Demonstration und Bewertung praktischer Fertigkeiten durch das neue Prüfungsformat als eingeschränkt. Dennoch könnten Tele-OSCEs eine sinnvolle Alternative darstellen und Studierenden die Möglichkeit zu geben, ihre zahnmedizinische Ausbildung in Zeiten von Kontaktrestriktionen fortzusetzen.

## 1. Introduction

Due to the coronavirus pandemic, many essential parts of medical education had to be paused. The necessity to limit students’ patient encounters and interactions with each other down to a minimum had and still has a tremendous effect on medical education [1]. 

Dental education in particular was drastically affected by these restrictions since the profession necessarily involves close examination as well as diagnostic and therapeutic interventions in the naso-oro-pharyngeal region, thus dentists are most susceptible to get infected with the corona virus [2]. A recent survey administered by the Association of Dental Education in Europe (ADEE) has captured the initial response of European dental schools to the coronavirus pandemic and pointed out the need for more research on distant learning methods [3]. In this context, dental schools worldwide are facing an inevitable change from the traditional chair-side teaching to virtual solutions such as tele-dentistry [4] or the use of virtual patients [5]. Although there can't be an exact replacement, telemedicine has proven to be an unique opportunity to bridge this current gap [6]. According to Sharma et al. there are a number of suggested skills that can be effectively integrated into medical and dental curricula using telemedicine. These include but are not limited to the following: communication, physical examination professionalism and technological literacy [7]. 

Tele-dentistry as a subunit of telemedicine is found to be comparable to real-time consultation in areas with limited access to facilities, in schoolchildren and in long-term-healthcare facilities [8]. Tele-consultation and tele-diagnosis have been rated positively and beneficial for dental care as well [8]. In terms of medical school training and examinations, telemedicine has already found its application in zero-patient contact virtual practical exit examinations for orthopaedic residents or running virtual objective structured clinical examination (OSCE) cases [9], [10]9. In a recent study by Pante et al. the implementation of a 6-station virtual OSCE was described and evaluated within a German post-graduate masters program (MME). The authors found that the virtual OSCE was well realizable in terms of time and organisation and was evaluated positively by the participants [11]. 

For dental education, there has been one pilot study that described the implementation of a virtual OSCE (VOSCE) in paediatric dentistry and orthodontics that worked well for both students and examiners. In this 10-station VOSCE Donn et al. used the video-call software zoom^®^ with its break-out room function to switch students from OSCE station to OSCE station. However, this required additional staff (lead host and lead invigilator) and examination time. The examined skills were reduced to communication and consultation since no standardized patients took part in this VOSCE. Students and examiners both evaluated the VOSCE favourably, however it was not investigated whether the new examination format would have an influence on student performance [12]. 

Therefore, the primary aim of the present study is to assess the feasibility and acceptance of a newly developed Tele-OSCE for dental students and examiners. We piloted a 3-station Tele-OSCE within oral and maxillofacil surgery (OMF) curriculum using the video-call software zoom^®^ and evaluated its acceptance among students and examiners. Furthermore, student performance was measured using standardized checklists and compared to a previous OMF OSCE which was conducted under normal circumstances in presence. A secondary aim was to compare both examination formats (Tele-OSCE and presence OSCE) regarding student performance. Our null-hypothesis was, that both examination formats would lead to different student performance. 

## 2. Methods

### 2.1. Ethics approval and consent to participate

The study was reviewed by the Ethical Commission of the University Hospital Frankfurt (Goethe University) and it was stated no further ethical approval was required. The study was conducted according to the Declaration of Helsinki [13]. Participation was voluntarily. All study participants gave their written informed consent prior to participation, which they could withdraw at any time. 

#### 2.2. Study participants

Study participants (study group: n=34, f=25, m=9; control group: n=32, f=21, m=11) were fourth-year dentistry students (in a five-year curriculum) in the period of 2021 attending a compulsory internship, which includes a five-day rotation through every section of the Department of Oral, Cranio-Maxillofacial and Facial Plastic Surgery, i.e. the operative room, the outpatient clinic or the emergency department. Before starting their rotation, students have to complete a practical skills training. In this practical skills training students were taught the most common reasons for a OMF consultation in a problem-orientated learning style and learned how to perform practical OMF skills (i.e. a structured facial examination, placing an “Ernst”-ligature or placing an i.v. catheter) in small groups up to six students [14]. The training lasted for 4 hours and was conducted by an experienced OMF surgeons who received a standardized blueprint about the practical skills training and its learning objectives beforehand. Both, the study- and the control-group, had to participate in the “practical skills training” in presence. Moreover, the study included nine board certified OMF Surgeons, with extensive experience in examining OSCEs, who served as examiners in the formative OMF OSCE at the end of the internship. 

#### 2.3. Study conduction

**Figure 1:**
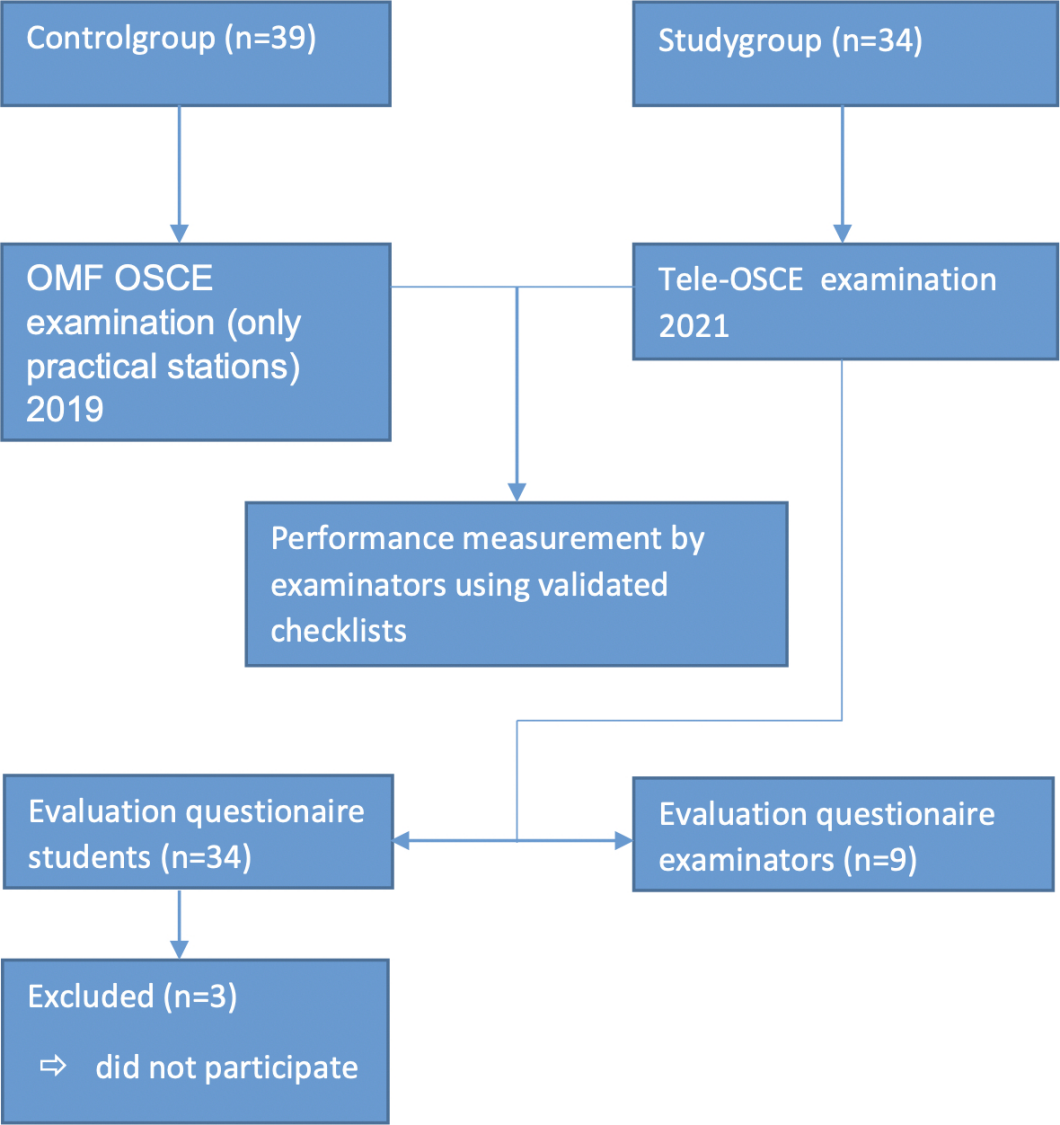
Study design

The study took place within the formative OMF OSCE seven weeks ± 1,5 weeks after the OMF internship. Under non-pandemic conditions, this OSCE is composed of eight five-minute stations, four of them verifying theoretical skills using multiple-choice tests and four of them assessing practical skills. Students rotate between theoretical and practical stations for which they have 5 minutes to complete. Frequently examined skills include i.e. the performance of a structured facial examination, management and consulting stations regarding frequent OMF consultations like squamous cell carcinomas, traumas of the facial skeleton or infections of the face and the oral cavity [15]. Due to the pandemic situation, the examination couldn’t take place under normal conditions. Therefore, a reduced three-station Tele-OSCE was implemented and piloted to formatively assess students' competencies (see figure 1 (Fig. 1)). 

Prior to the Tele-OSCE, examiners were given instructions on how to use the video-call software Zoom (Zoom video communications, San José, California, USA) and were provided with a standardized Power Point^®^ presentation containing the OSCE station scenarios. Moreover, examiners received previously validated and published OSCE Checklists [15] for each OSCE station and an Tele-OSCE time schedule with the names and e-mail addresses of each student that were assigned to one of the examiners before the study conduction (see attachment 1 ). 

**Figure 2:**
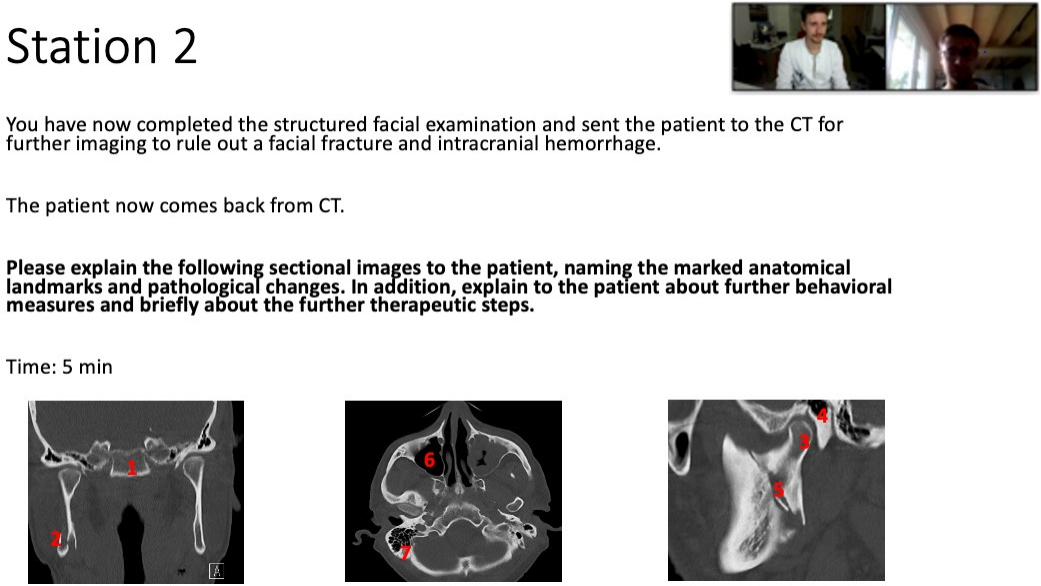
Exemplary interaction between examiner and student in the OSCE station “management mandibular fracture” via zoom^®^, Power Point presentation with precise working assignment presented to students using the screen share function for one minute. Students had five minutes to complete the OSCE assignment. Each examination took 18 minutes. The remaining 12 minutes were used for feedback and evaluation of the new Tele-OSCE format.”

On the day of the study conduction, students received an e-mail invitation for their scheduled examination and met up with the examiners in a private online-meeting to carry out the Tele-OSCE examination. Each online meeting was set up for 30 min. First, students received a short explanation about the upcoming examination by the examiner, then they completed three OSCE stations, namely the performance of a structured facial examination, the management of a mandibular fracture as well as the management of an oral squamous cell carcinoma with the same examiner. For each OSCE station, students received a precise working assignment which was presented for one minute via the screen share function (see figure 2 (Fig. 2)). Afterwards, students had five minutes to complete the OSCE assignment. Each examination took 18 minutes. The remaining 12 minutes were used for feedback and evaluation of the new Tele-OSCE format. Due to the new setting, the examination itself had to be slightly adapted, i.e. x-rays normally used within the station “management mandibular fracture” were presented via the screen share function using standardized Power Point® slides. Moreover, students were told to correctly describe in their own words how they would perform a structured facial examination step-by-step since no patient actor was present. After completion, every examiner met up with a new student and repeated the examination. In case of technical problems, a special Zoom^®^ room was provided for students and examiners. Every OMF Surgeon involved examined between three to four students. In total, the Tele-OSCE lasted for two hours. 

#### 2.4. Performance measurement

Students' performance was measured using previously validated OSCE Checklists [12]. Prior to the study all examiners received an educational course as calibration and to gain experience using the OSCE Checklists (see attachment 1 ). In addition, the content validity was ensured through the creation as part of an expert workshop with didactic and surgical experts as well as through the repeated application and adaption in the context of previous studies [14], [15], [16], [17] and OSCE exams. 

#### 2.5. Tele-OSCE evaluation

Prior to the examination, two evaluation questionnaires (see table 1 (Tab. 1) and table 2 (Tab. 2)) (one for the students and one for the examiners) were designed and concerted by an expert group based on a literature review. The items were tested regarding comprehensibility by a group of medical education specialists who were not involved in designing the questionnaires. Based on the answers of the medical education specialists, the questionnaires were edited. Each questionnaire consisted of 10 statements, five of which were concurrent for both groups. The remaining five questions were group-specific. The questions were answered by both groups after the OSCE by using a 5-point Likert-scale ((1) strongly agree; (2) agree; (3) neither agree nor disagree; (4) disagree; (5) strongly disagree). Moreover, the questionnaire contained free-text commentaries for written feedback. 

#### 2.6. Statistical analysis 

Microsoft Office 2016 (^©^ Microsoft Corporation, Redmond, USA) for Mac and SPSS Statistics version 19 (IBM, Armonk, USA) were used for the statistical analysis. Student performance was tested for normal Gaussian distribution using the Shapiro-Wilk normality test. Furthermore, the sample size needed was calculated a priori using the software G*power (Version 3.1.9.6) with the following settings: tails (1), distribution (logistic), effect size (0.5), alpha (0.05), 1-beta error probability (0.8). Examination results from the Tele-OSCE were compared to the OMF OSCE examination results (only practical stations) in the summer term 2019 to analyse whether the new examination format would have any influence on student performance. To test for significant differences, the Mann-Whitney U-test was used, since data were not normally distributed. Data was presented as mean ± standard deviation. 

## 3. Results

### 3.1. Statistical power, study participants and study conduction

The calculated sample sizes for the control- and study group was 51 participants per group. Sixty-six dental students (study group: f=25 m=9, 100% of the semester, summer term 2021, control group: f=21, m=11, 100% of the semester, winter term 2019) and nine board certified OMF Surgeons (f=3, m=6, 42% of the Department) took part in the study. Due to a technical problem (instable internet connection) at the beginning of the Tele-OSCE, eight of 34 students had to postpone their examination appointment by one hour to keep up with the planned schedule. After switching the examiners internet connection, no other technical problems occurred and the conduction of the Tele-OSCE went fluently and was feasible within the given timeframe. 

#### 3.2. Performance measurement

Compared to the OMF OSCE examination results from the summer term 2019, there were no significant (p=0,53; CG (control group): Av=75,35%±7,7%; SG (study group): Av=72,59%±14,0%) differences in overall student performance in the Tele-OSCE. In the OSCE stations’ “structured facial examination” (p=0,25; CG: Av=77,28%±10,2%; SG: Av=79,76%±14,7%) and “management of a squamous cell carcinoma” (p=0,36; CG: Av=62%±11,2%; SG: Av=65,09%±17,6%) no significant differences in student performance were observed compared to the OMF OSCE in 2019. However, in the OSCE station “management mandibular fracture”, students achieved significantly less points (p<0,01; CG: Av=86,81%±11,6%; SG: Av=72,97%±14,2%) than in 2019. 

#### 3.3. Student evaluation

31 out of 34 (91 %) students (study group) completed the evaluation questionnaire (see table 2 (Tab. 2)). The majority of students felt that the demonstration of their performance was possible without any problems and did not fear to be misjudged due to the new format. Moreover, the demonstration of anamnestic and consultation competencies was rated as unproblematic by students. However, the demonstration of practical skills, such as the performance of a structured facial examination, was seen as more problematic. The video- and audio-quality were overall rated as very good and most students stated that they would like to participate in future Tele-OSCEs. 

In the free-text commentaries, students commended the “good atmosphere” (n=4), the “well-structured time schedule” (n=1), and the “given time for direct verbal feedback” by the examiner after the examination (n=3). However, some students criticized “technical problems at the beginning” of the Tele-OSCE (n=3) and would have preferred models or phantoms to better demonstrate their facial examination skills (“use of phantoms”, n=1). 

#### 3.4. Examiner evaluation

All nine examiners completed the evaluation questionnaire (see table 3 (Tab. 3)). Like the student evaluation, most examiners felt that, overall, student assessment was possible without any problems, especially for anamnestic and consultation competencies, but expressed impeded assessment of practical skills within the Tele-OSCE format. Examiners rated the audio- and video signal as very good, but also were critical regarding the technical implementation and conduction of the Tele-OSCE. Nevertheless, most examiners stated that they would like to participate in future Tele-OSCE examinations.

In the free text commentaries, examiners praised the possibility to “carry out the examination from home” without any disruptions due to clinical duties (n=1). Examiners also commended that they could “examine one student through the entire Tele-OSCE and, thus, had a better impression of the student’s level of competence” (n=1). Similar to the students, examiners also would have “preferred phantom models or standardized patients for the demonstration of facial examination skills” (n=2). 

## 4. Discussion

The current study investigated the development and curricular integration of a virtual Tele-OSCE examination in Oral- and Maxillofacial Surgery as an alternative to the traditional OSCE for dental students at our institution due to corona virus contact restrictions. Overall, our results show that the implementation of the OSCE was (apart from minor technical problems at the beginning of the examination) feasible within the given timeframe and was well-accepted by students and by examiners. 

Interestingly, no significant differences in student performance were found for the OSCE stations “structured facial examination”, even though both students and examiners expressed difficulties in demonstrating and rating a purely practical skill. An explanation for the inferior student performance in the OSCE station “management mandibular fracture” compared to non-pandemic examinations might be limited screen size of the tablets and smartphones which many students used to take part in the examination. This might have limited students to correctly allocate anatomical landmarks and pathologies in the presented CT scans of the OSCE station (see figure 2 (Fig. 2)) and should be considered for future Tele-OSCE examinations. 

There have been other studies that sought to investigate the use of tele-medicine as an alternative teaching and examination format during the coronavirus pandemic. Harendza et al. used tele-medicine within a newly designed competence-based training, including a consultation hour, with simulated patients for final year medical students and found similar satisfaction levels with the training compared to non-pandemic years [18]. In the present study, simulated patients or phantom models were not included due to capacity reasons since eight OSCE examinations were conducted parallel to stay within the given timeframe of two hours. This might have led to the rather weak evaluation regarding the demonstration of practical skills within the Tele-OSCE by students and could be viewed as a limitation of the study. On the other hand, our setting allowed a more genuine evaluation of the students’ acceptance with the newly established examination format. 

Sartori et al. developed a Tele-OSCE scenario as part of a multi-station OSCE simulating a remote encounter between a resident and a recently discharged standardized patient for internal medicine residents as well as a concordant assessment tool [9]. Similar to the present study, they found weak areas in the examination format regarding the demonstration of a virtual physical examination, which might be due to the missing simulated patients or the missing physical encounter between examiner and simulated patient. In similar study Pante et al. described the implementation of a 6-station virtual OSCE within a German post-graduate masters program (MME). The authors also used breakout-rooms into which the participants rotated independently. They showed that a telemedical OSCE is technically feasible, but they concluded that further strategies must be developed i.e new checklists for the rating of non-verbal communication or special SP training to adapt the OSCE to the new digital format. Although, participants stated that the creation and participation was not easy to them, like in our study, they considered the implementation of digital examination formats to be important in the future [11]. 

Regarding dental education, Donn et al. piloted a virtual dental OSCE (VOSCE) for undergraduate dental students and gave a comprehensive exemplary description of how to set up a VOSCE [12]. The newly piloted VOSCE was rated favourably by undergraduate dental students and staff. In contrast to the present study, this VOSCE used multiple Zoom breakout rooms for the OSCE stations (12 minutes per station) and waiting rooms for students and examiners. This enabled students and examiners to switch stations within the VOSCE and might have led to a more balanced assessment of student performance since every student was assessed by multiple examiners. On the other hand, the stated that the use of break-out rooms was very staff- and time-intensive since the simultaneous moving of students required a lead- and host-invigilator and additional time for room switching. In the present study, we decided not to use breakout rooms, which simplified the Tele-OSCE workflow, limited the potential for failure and allowed us to save personnel which would have been necessary to allocate students and examiners to their respective rooms. 

## 5. Limitations and strengths

The small sample sizes of the study- and control group and hence low statistical power might be a limitation to the conclusions drawn from the comparison of performances between both examinations formats. On the other hand, this study was conducted in an “in vivo” examination setting with a 100 % participation rate and for the first time analysed student performance in a Tele-OSCE compared to non-pandemic years and evaluated the new examination format from a student and examiner perspective. Compared to previous OMF OSCE examinations no additional personnel-hours were necessary, since the entire OSCE was conducted simultaneously by the 8 examiners, however this required the conduction after working-hours. Another positive aspect of the 30 minutes timeframe for the completion of the three OMF stations was the possibility of individual feedback to the students, especially since prior studies have shown the importance of a structured feedback within a surgical OSCE [19]. It remains unclear whether the different times of interventions (2019 and 2021) might have had an influence on the comparability of the study- and control-group, however both groups received the practical skills training in presence prior to the OMF OSCE participation. 

## 6. Conclusion

The curricular implementation of a Tele-OSCE in Oral- and Maxillofacial Surgery seems feasible, is overall well-accepted by students and examiners and does not lead to significant lower overall student performance. Tele-dentistry offers a safe alternative to enable students to complete their curricular training in times of contact restrictions. Moreover, the integration of virtual examination and teaching formats might be a useful and cost-effective alternative to traditional teaching formats in dentistry since this study and other studies showed that tele-dentistry is suitable for OSCE examinations and the mediation of other skills like patient communication, physical examination, professionalism and technological literacy [7]. To further increase acceptance, virtual teaching and examination formats should be integrated early in medical and dental curricula. 

## Abbreviations

- OSCE: objective structured clinical examination
- OMF: oral and maxillofacial

## Competing interests

The authors declare that they have no competing interests. 

## Supplementary Material

OSCE checklists for the three stations

## Figures and Tables

**Table 1:**
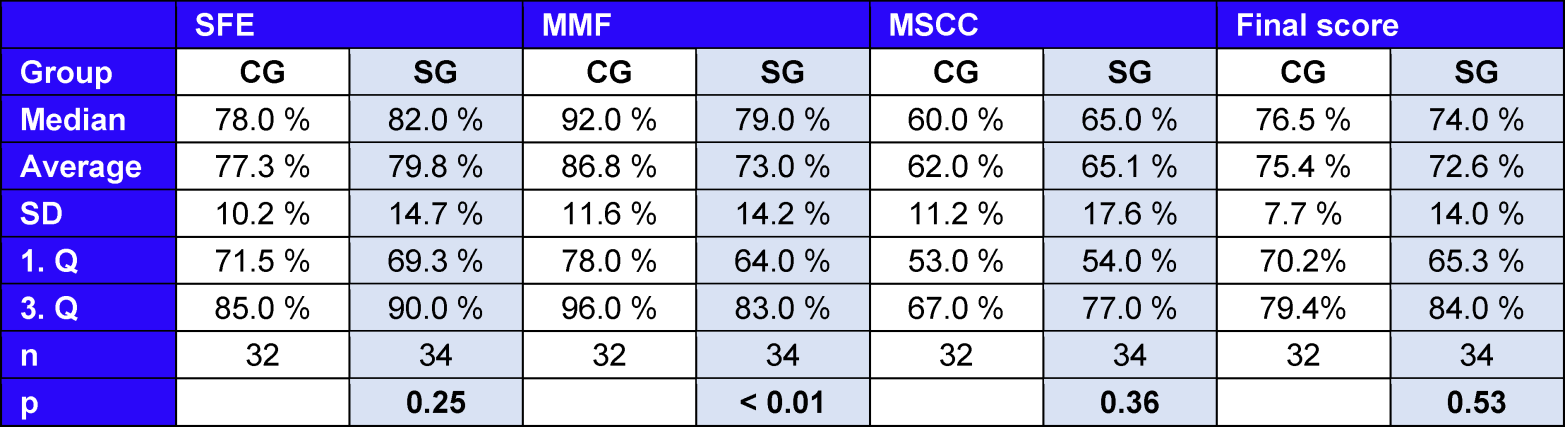
Performance measurement of the study group (SG) and control group (CG) for the structural facial examination (SFE), management mandibular fracture (MMF), management of a squamous cell carcinoma (MSCC) and the final score including average, median, standard deviation (SD) and sample size (n) and significance test of student performance using the Mann-Wilcoxon-Whitney-U-Test

**Table 2:**
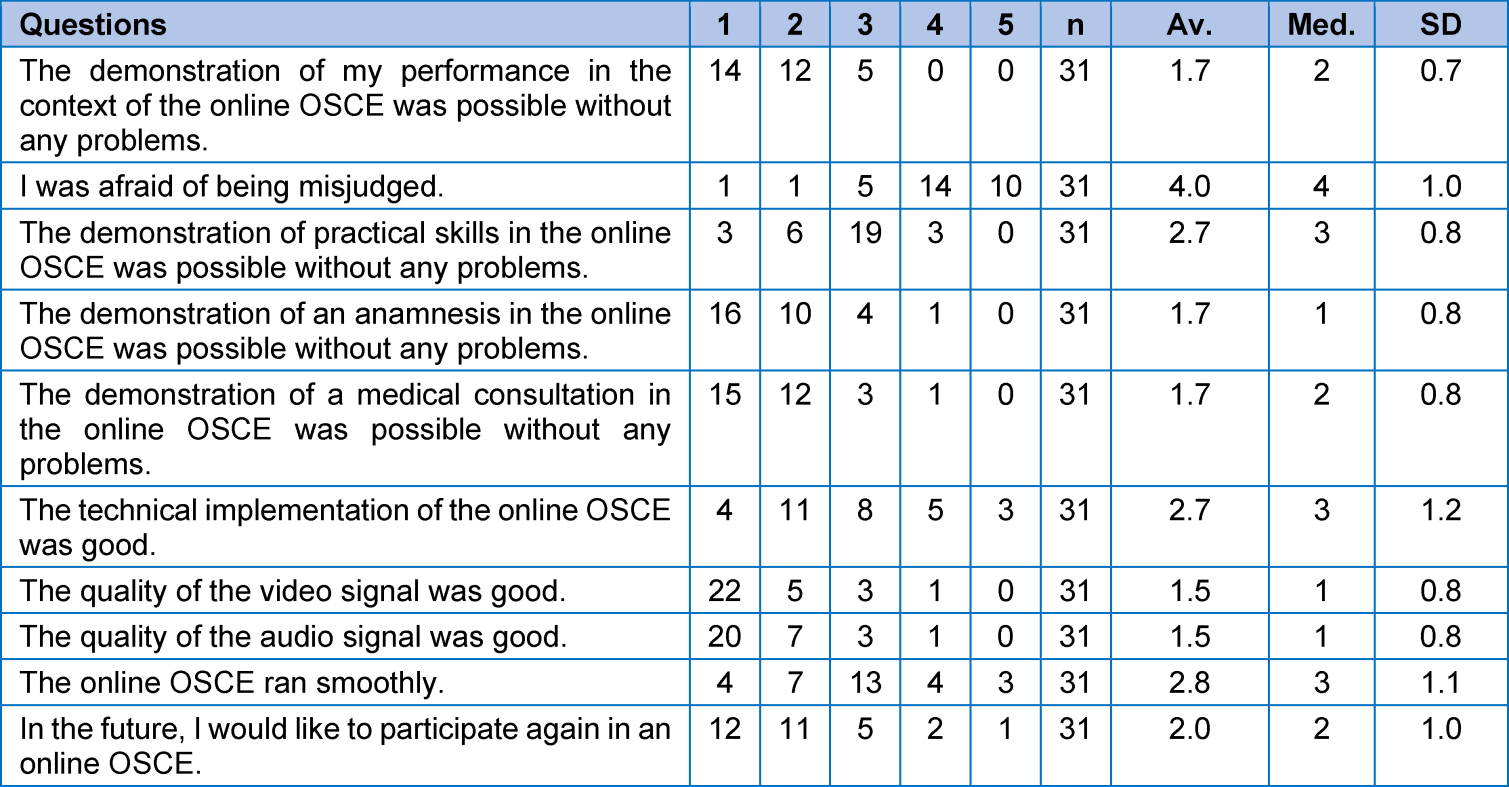
Student evaluation questionaire using a 5-point Likert-scale ((1) Strongly agree; (2) Agree; (3) Neither agree nor disagree; (4) Disagree; (5) Strongly disagree). (Av.=average, Med.=median, SD=standard deviation)

**Table 3:**
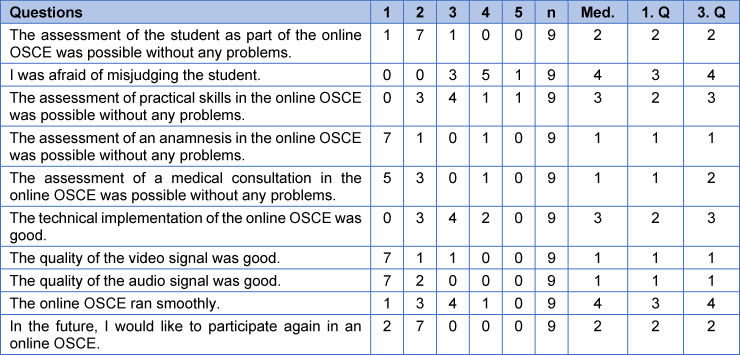
Examiner evaluation questionnaire using a 5-point Likert-scale ((1) Strongly agree; (2) Agree; (3) Neither agree nor disagree; (4) Disagree; (5) Strongly disagree). (Av.=average, Med.=Median, SD=standard deviation)
