# Supplementary material for: Implementation and evaluation of a Tele-OSCE in oral and maxillofacial surgery – a pilot report
Source: GMS J Med Educ. 2022 Nov 15;39(5):Doc50. doi: 10.3205/zma001571 (PMC9733482; doi:10.3205/zma001571)
Supplement: OSCE checklists for the three stations [file JME-39-50-s-001.pdf]

Attachment 1: OSCE checklists for the three stations: (A) management mandibular fracture (MMF), (B) structural facial examination (SFE), (C) management of a squamous cell carcinoma (MSCC). A trinary system was used to to rate each item. Two point were given for the complete and correct execution, one point for the incomplete or not entirely correct execution, zero point for no execution at all.

A

Item	Given points	Max Points
Introduction (name, function, intention)		2
Correctly naming radiological imaging		2
Correctly naming anatomical structure Nr. 1 (porus acusticus ext.)		2
Correctly naming anatomical structure Nr. 2 (maxillary sinus)		2
Correctly naming anatomical structure Nr. 3 (mentum)		2
Correctly naming anatomical structure Nr. 4 (septum nasi)		2
Correctly naming anatomical structure Nr. 5 (foramen mentale)		2
Finding and naming pathology Nr. 1 (paramedian fracture)		2
Finding and naming pathology Nr. 2 (collum mandibulae fracture)		2
Correctly explaining the further procedure (antibiotics, cooling, non-solid food)		2
Correctly naming occlusion establishing procedures (temporary intermaxillary fixation)		2
Indication and explanation of surgical treatment with microtitanium plates		2
**Total**		**24**

B

Item	Given points	Max Points
Introduction (name, function, intention)		2
Correct inspection of the face		2
Correct examination of the visus		2
Correct examination of the pupils		2
Correct examination of double images		2
Correct examination of the trigemal nerve N.		2
Correct examination of the facial nerv		2
Correct examination of the skull		2
Correct examination of the orbital rim		2
Correct examination of the malar bone		2
Correct examination of LeFort 1, 2 and 3		6
Correct examination of the Nasal bone (motility, breathing, septal hematoma)		6
Correct examination of the caput (mandible)		2
Correct examination of the corpus (mandible)		2
Correct examination of the compression pain (mandible)		2
Correct examination of the motility (mandible)		2
Correct examination of the oral cavity (inspection, occlusion, loose teeth, mouth opening)		8
**Total**		**48**

C

Item	Given points	Max Points
Introduction (name, function, intention)		2
Correct explanation of the pathological finding		
- squamous cell carcinoma		2
- dignity		2
Correct explanation of the necessary further diagnostics (Staging):		
- CT scan thorax and abdomen		2
- MRI head and neck		2
- Sonography head and abdomen (alternative)		2
- X-ray thorax (alternative)		2
Correct explanation of the further surgical procedures		
- Surgical excision of the tumour		2
- Neck dissection		2
- Tracheotomy		2
- reconstruction with free flap		2
- stationary		2
- rehabilitation after surgery		2
Correct explanation of alternatives to surgery		
- radiotherapy/brachytherapy		2
- chemotherapie		2
**Total**		**30**
